# Transfection of the BDNF Gene in the Surviving Dopamine Neurons in Conjunction with Continuous Administration of Pramipexole Restores Normal Motor Behavior in a Bilateral Rat Model of Parkinson's Disease

**DOI:** 10.1155/2024/3885451

**Published:** 2024-02-21

**Authors:** Alina Benítez-Castañeda, Verónica Anaya-Martínez, Armando de Jesús Espadas-Alvarez, Ana Luisa Gutierrez-Váldez, Luis Fernando Razgado-Hernández, Patricia Emmanuelle Reyna-Velazquez, Liz Quintero-Macias, Daniel Martínez-Fong, Benjamín Florán-Garduño, Jorge Aceves

**Affiliations:** ^1^Center for Research and Advanced Studies of the National Polytechnic Institute, Mexico City, Mexico; ^2^Center for Research in Health Sciences, North University Anahuac, Mexico City, Mexico; ^3^Neuromorphology Laboratory, National Autonomous University, Iztacala Campus, Mexico City, Mexico

## Abstract

In Parkinson's disease (PD), progressive degeneration of nigrostriatal innervation leads to atrophy and loss of dendritic spines of striatal medium spiny neurons (MSNs). The loss disrupts corticostriatal transmission, impairs motor behavior, and produces nonmotor symptoms. Nigral neurons express brain-derived neurotropic factor (BDNF) and dopamine D3 receptors, both protecting the dopamine neurons and the spines of MSNs. To restore motor and nonmotor symptoms to normality, we assessed a combined therapy in a bilateral rat Parkinson's model, with only 30% of surviving neurons. The preferential D3 agonist pramipexole (PPX) was infused for four ½ months via mini-osmotic pumps and one month after PPX initiation; the BDNF-gene was transfected into the surviving nigral cells using the nonviral transfection NTS-polyplex vector. Overexpression of the BDNF-gene associated with continuous PPX infusion restored motor coordination, balance, normal gait, and working memory. Recovery was also related to the restoration of the average number of dendritic spines of the striatal projection neurons and the number of TH-positive neurons of the substantia nigra and ventral tegmental area. These positive results could pave the way for further clinical research into this promising therapy.

## 1. Introduction

In Parkinson's disease (PD), the progressive degeneration of the dopamine neurons of the substantia nigra pars compacta (SNc) triggers the deafferentation of the striatum, which leads to the characteristic motor dysfunctions of the disease. The critical motor dysfunctions are the loss of normal gait, bradykinesia, muscle rigidity, and reduced motor coordination and balance. The disease also produces nonmotor alterations, such as anxiety, depression, and reduced memory and cognitive abilities. The clinical deterioration reflects ongoing nigrostriatal dopaminergic degeneration. BDNF plays a critical role in the physiology and pathophysiology of the basal ganglia. Sustained expression of BDNF is essential for preserving the morphology of dendrites and synapses of medium spiny neurons (MSNs) [1, 2]. PD leads to a profound and selective decrease in the length and arborization of dendrites and a loss of the dendritic spines in MSNs [3–5]. Loss of dendritic spines also occurs in PD models [6, 7] and is the cause of loss of corticostriatal synapses [8, 9], the basis of the pathophysiology of the disease.

As BDNF, dopamine has not only a functional but also a trophic effect [10, 11]. The trophic effect is mediated, in part, by D3 receptors, whose activation is essential for the survival and protection of the dopamine neurons [12, 13] and the preservation of their dendrite arborization [14]. The activation of D3 receptors by their agonists and TrkB receptors by BDNF occurs through similar intracellular pathways to exert their neurotrophic effects [15]. There are reports of crosspotentiation between dopamine receptors and BDNF receptor activation. Zhou et al. reported an increase in the number of dopamine cells in the cerebral cortex of rat embryos in culture (E12-14); the BDNF treatment significantly increased (up to 17-fold) the number of dopamine neurons induced respect dopamine alone [10]. Furthermore, a synergistic effect of BDNF and dopamine induces the dopaminergic phenotype in the rat embryonic cerebrum [16].

Previously [17], we showed that continuous intraperitoneal infusion of the preferential D3 agonist 7-hydroxy-N, N-di-n-propy1-2-aminotetralin (7-OH-DPAT), associated with the selective BDNF-gene transfection into the surviving dopamine neurons of rats with a unilateral intrastriatal 6-OHDA lesion of the nigrostriatal innervation (50% of surviving neurons), restored the TH-positive dopamine neurons of the SNc, the dendritic spines of the striatal neurons as well as the motor behavior (gait, motor coordination, and muscular rigidity). The recovery appeared to be permanent because it was still present two months after cessation of treatment and was associated with neuroanatomical restoration of nigrostriatal innervation. Since BDNF-gene transfection is selective for the dopamine neurons, this approach depends on the number of surviving dopamine neurons before treatment. To translate this therapy into the clinic is crucial to test the effectiveness of the combined treatment in a more advanced PD model, namely, rats with only 30% of tyrosine hydroxylase positive neurons (TH+) in the substantia nigra pars compacta (SNc) of both sides. In the present study, we used pramipexole (PPX), the D3 agonist widely used in the clinic, to treat the signs and symptoms of idiopathic PD instead of 7-OHDPAT and combined it with BDNF-gene transfection. The PPX was administered continuously via subcutaneously implanted osmotic pumps. To directly test the effect of the combined treatment, we compare the therapy with the normal and denervated states. Finally, we also examined whether the new combination treatment restored working memory, dendritic spines of striatal neurons, and TH-positive neurons in both the SNc and VTA, in addition to motor behavior.

## 2. Materials and Methods

**Figure 1:**
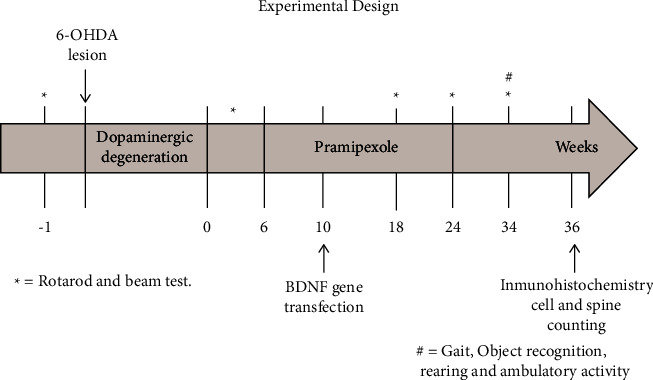
Experimental protocol. 6-OHDA was injected into three sites in the right striatum and one week later into the contralateral striatum (see Methods and Materials for coordinates). PPX was continuously infused via subcutaneously implanted osmotic pumps. The BDNF gene was transfected once one month after the start of the PPX infusion. The final rotarod and beam test was performed two and a half months after the end of the PPX infusion.

All experimental procedures strictly followed the current Mexican legislation, NOM-062-ZOO-1999 and NOM-087-ECOL-1995 (SAGARPA), based on the Guide for the Care and Use of Laboratory Animals, NRC. The Animal Care and Use Committee of the Center for Research and Advanced Studies (CINVESTAV) approved our procedures for animal use (protocol #0240-17). Every effort was made to minimize animal suffering. Thirty rats weighing 250 g and six weeks old at the beginning of the study were chosen. They were kept under an inverted 12 : 12 dark-light cycle and had ad libitum access to food and water. Beam and Rotarod tests were performed before the lesion as initial parameters of the locomotor activity of each rat to monitor their performance during the study. We used the rotarod test as the discriminative task before and after one month (4 weeks of dopaminergic degeneration) of the 6-OHDA lesion; we selected only those rats with 50% loss of their average rotarod performance. Those tests took two weeks to be completed (Figure 1: time “0”). We assembled the groups as follows: We selected fourteen lesioned rats, fulfilling the requirement for the rotarod performance, and divided them into two groups of 7 rats each: (1) rats treated with the D3 receptor agonist, PPX, associated with the BDNF-gene transfection (PPX + BDNF rats), (2) untreated lesioned rats (Untreated rats). In addition, we had a group of healthy, control rats (Intact rats). After rotarod evaluation, the PPX infusion was started into those rats that fulfilled the acceptance criteria. We evaluated motor performance (rotarod and beam test) every one½ months during treatment at 1.5, 3, and 4.5 months and two months after withdrawal of the PPX infusion to assess the time course of the treatment effect and the persistence of the effect. In addition, naïve rats were used to evaluate the loss of locomotor activity due to aging (Figure 1).

### 2.1. 6-OHDA Lesion

Male adult Wistar rats were anesthetized with ketamine (Ketamine-Pet Laboratory Aranda 75 mg/kg) and procin 2% (Xylazine, Pisa, 5 mg/kg) intraperitoneally. They were mounted on a stereotaxic apparatus (Kopf model 201025R; Tujunga, CA, USA), and then 6-OHDA (6-Hydroxydopamine hydrochloride, H4381, Sigma Aldrich, Saint Louis, MO, USA) was slowly injected (1 *μ*l/5 min) via a 30G injection needle connected through a polyethylene tube to a ten *μ*l Hamilton syringe driven by a Micro-Syringe Pump Controller (World Precision Instruments). The coordinates for the striatum were obtained from the stereotaxic Atlas of Paxinos and Watson [18]. In the first step, the 6-OHDA was injected into three striatal sites (7 *μ*g/site) according to the following coordinates [19]: AP = +1.9, ML = ±2.5, DV = +5; AP = +0.8, ML = ±2.9, DV = +4; and AP = −0.5, ML: ±5.2, DV = +5 mm. The references for the coordinates were the bregma (AP), the intraparietal suture (ML), and the dura mater (DV). One week later, the 6-OHDA was injected into the contralateral striatum using mirror coordinates in a second surgery. 6-OHDA was dissolved in a saline solution containing 0.1% ascorbic acid, kept on ice (4°C), and protected from light to minimize oxidation. Upon completion of the injection, the needle remained in its place for 3 min to prevent backflow and allow diffusion of the 6-OHDA. After the scalp wound was sutured, we placed the rats in separate cages and treated them with terramycin 2 g/L dissolved in drinking water for two weeks. Immediately after the procedure, the rats were quickly and gently removed from the stereotaxic apparatus and placed supine in another chamber for recovery.

### 2.2. Pramipexole Administration

We administered the dopamine D3 agonist PPX (pramipexole dihydrochloride PHR1598; Sigma-Aldrich; Saint Louis, MO, USA) through the mini-osmotic pump model 2006 (Alzet; Cupertino, CA, USA) at a dose of 0.5 mg/kg/day. We implanted the pumps subcutaneously under anesthesia and aseptic conditions to infuse the D3 agonist for four ½ months and replaced the pumps every one ½ month. Treatment begins six weeks after the lesion of the second striatum and lasts for 18 weeks.

### 2.3. BDNF-Gene Flag Transfection by the NTS-Polyplex

The plasmid phDAT-rBDNF-flag (10.511 kbp) encoding the rat BDNF-flag was transfected bilaterally into the substantia nigra compacta using the neurotensin (NTS)-polyplex nano vector [20], a nonviral gene transfer system selective for dopamine neurons endocytosed via the high-affinity neurotensin receptor present only in the dopamine neurons [21]. The BDNF-flag gene expression is controlled by the human dopamine transporter gene promoter (hDAT) [19, 21, 22]. The optimum molar ratios of NTS-polyplex components were 30 nM plasmid DNA, 30 *μ*M karyophilic peptide, and 1.17 *μ*M NTS-FP-PLL (a conjugate of NTS, a fusogenic peptide and poly-L-lysine) [19, 23]. For these molar ratios, the NTS concentration was 1.17 *μ*M, as determined previously with 125I-NTS [19]. Considering this NTS concentration and the injection volume (2 *μ*l), the NTS-polyplex dose was 2.34 pmol for rats of 550 g of mean body weight. By the amount of plasmid DNA, the dose was 419.6 ng of phDAT-BDNF-flag [19, 24]. The surgical procedure for transfection was like a 6-OHDA injection. We performed the transfection one month after the start of the pramipexole infusion [25]. We injected two microliters of NTS-nano vector carrying the plasmid phDAT-rBDNF-flag slowly (0.1 *μ*l/min) into the border of the SNc to avoid damaging the neurons. The injection coordinates were AP = −5.6, ML = ±1.9, and DV = −7.1 [21]. The AP and ML coordinates were from bregma, according to Paxinos and Watson [18].

### 2.4. Beam Test

After training the animals for four days, we assessed balance and motor coordination to cross five times a 2 m long wooden beam flat-surfaced with a slope of 17°. On the first day, we used an 18 mm wide beam for adaptation, and another 12 mm wide was replaced from the second to the fourth day. We assessed the performance of the animals on the fifth day by video recording them as they crossed the 12 mm and a novel 6 mm beam and quantified the crossing time.

### 2.5. Rotarod

This test evaluates motor coordination, balance, and motor learning [26, 27]. The motor performance of rodents in the rotarod allows the evaluation of the loss or recovery of the nigrostriatal innervation in rodent PD models [27]. The rotarod consists of a four-lane rotating rod (diameter 6 cm) and infrared beams to detect the time of the fall. The rat's body is placed perpendicular to the axis of rotation, with the head against the direction of rotation, so the animal must move forward to stay on the road. We assessed the rat permanence on the rod at a constant definite speed. We trained the rats twice on the rotarod at 5 and 10 rpm for two minutes for three consecutive days before evaluating their performance. In the testing session, we placed rats on the rod and assessed their permanence at 10, 15, 20, and 25 rpm for a maximum of 2 min at each speed. For each animal, we calculated the overall rotarod performance (ORP) as the area under the curve of the plot time-on-the-rod against rotation speed [27]. We video-recorded all rats while they remained on the rod to assess the recovery of motor coordination and balance.

### 2.6. Gait Analysis

We recorded the unrestrained gait of rats on a transparent acrylic runway (track) (17 cm high, 15 cm wide, and 170 cm long, with a dark compartment at its end for sheltering). The runway was 150 cm above floor level. We used two DSC-W630 Sony Cyber-shot cameras, which were automatically synchronized, and placed one on each side at the runway's center. We determined the optical deformation of the image produced by the camera lens and corrected it using an acrylic box (5 cm × 5 cm) that served as a two-dimensional scale. We marked the iliac crest, the greater trochanter, the lateral malleolus, and the fifth metatarsal distal head with indelible ink as a reference for the analysis. We traced the marked points frame by frame, obtaining two-dimensional coordinates (*x*, *y*) using ImageJ software (NIH; Bethesda, MD, USA https://rsb.info.nih.gov/ij/). We analyzed the data using Microsoft Office Excel 2010 (Microsoft) and preassembled Excel sheets to model body segments as rigid straight lines between the marked points. We indirectly computed the knee position by superimposing two circles centered on hip and ankle pivots, with a radius of the length of the femur and tibia bones [28]. We reconstructed the kinematics of gait from changes in the marked points between consecutive frames, facilitating the generation of stick diagrams (superimposing modeled body segments of every frame) and spatial displacement plots. We calculated the angles and distances directly using the software. We constantly trained the rats to move along the runway for three consecutive days before the recording session. We recorded three satisfactory strides when the rat constantly walked through the runway's center.

### 2.7. Locomotor Activity, Spontaneous Ambulatory Activity, and Rearing

We determine locomotor activity in activity boxes with dark acrylic walls measuring 44 cm × 22 cm × 26 cm height equipped with sixteen infrared light beams positioned along the sidewalls that record the interruption of a beam as a measure of activity; two continuous interruptions assess displacement. We measured locomotor activity every minute for an hour as overall activity, measured the distance traveled, and counted the number of rearing events from the videos of the rats in the activity cages.

### 2.8. Object Recognition Test

We used the object recognition test [29] to assess working memory recovery. The apparatus was a square wooden box (45 cm/side, 50 cm height). We conducted the test in an open field in complete darkness. In the test trials, we placed two objects invariably in a symmetrical position about 5 cm away from the box wall. We used four objects with constant height and volume but different shapes and appearances. During habituation, we allowed the animals to explore the empty arena. We conducted the familiarization phase by placing individual rats in the field for 5 min, with two identical objects (A1 and A2) positioned in two adjacent corners. In a second session an hour later, the rats explored the open field arena for 2 minutes in the presence of one familiar (A1) and one novel (B). We chose the objects after assessing, in preliminary experiments, that they were equally preferred. We recorded all test periods and calculated the IRNO (index of recognition of a novel object) as the time of exploration of the unknown object minus the time of exploration of the familiar one divided by the total time of exploration (2 min). Finally, we cleaned the open-field arena and the objects with ethanol solution and replaced the arena bedding between trials.

### 2.9. Midbrain Tissue Slices

We euthanized the animals under anesthesia at the end of the experiment, 13 months after the 6-OHDA lesion. Next, we perfused the rats intracardially (120 mL of 0.9% saline solution, followed by 300 mL of 4% paraformaldehyde in PBS 0.1 M) using a peristaltic pump (Masterflex, Model 7535-10; Illinois et al.); finally, we removed the brains and stored them in 30% sucrose at 4°C for cryoprotection. We obtained coronal slices (30-*μ*m width) from the midbrain using a freezing sliding microtome (Leica; Heidelberg, Germany) and collected them sequentially into five glass vials containing phosphate-buffered saline (PBS 0.1 M, pH 7.4) so that each contained a complete representative sequential set of nigrostriatal innervation.

### 2.10. Immunofluorescence

We used double immunofluorescence against the flag epitope and tyrosine hydroxylase (TH) to detect the BDNF-gene expression in dopamine neurons of the SNc. First, we permeabilized the slices (0.5% Triton X-100 in PBS 4 times for 10 min at room temperature). We then replaced it with PBS with 0.5% SDS solution to recover the specific antigen. Afterward, we blocked unspecific binding sites and incubated the slices in PBS containing 0.5% Triton X-100 and 10% BSA. After washing twice for 5 min with 0.1 M PBS (30 min 37°C), we exposed the slices to the rabbit polyclonal anti-TH antibody (1/1000; Millipore; Billerica, MA, USA, Cat # AB152) and the mouse monoclonal antiflag M1284 antibody (1/300; Sigma-Aldrich Co.; St. et al., USA, Cat # AB924) in 0.1 M PBS/3% BSA, for 48 h in the dark (RT) and then washed three times with 0.1 M PBS for 5 min each. Afterward, we incubated the slices with a CY5-conjugated goat anti-rabbit IgG (1/200; Invitrogen-Thermo-Fisher Scientific, Waltham, MA USA, Cat # 81-6116) and an Alexa 488-conjugated sheep anti-mouse IgG (1/200, Invitrogen-Thermo-Fisher Scientific, Waltham, MA USA, Cat # A-11001) in 0.1 M PBS/3% BSA for two *h* at RT. After three washes with 0.1 M PBS (5 min each), we placed the slices on glass slides using VECTASHIELD (Vector Laboratories; Burlingame. CA, USA, Cat # H-1000) and protected them with coverslips. We detected the fluorescence using a confocal microscope (Leica, TSC SP8). Sequential slices every 150 *μ*m along with the rostrocaudal extension of the substantia nigra were scanned at 1 *μ*m optical thickness, 30–35 optical slices in total in the Z-series; then, we projected the integrated images onto a two-dimension plane. We overlapped the fluorescent images on the screen monitor using green for Alexa 488 and red for CY5. We obtained negative controls by omitting the primary antibody. We counted the TH (+) and BDNF-flag (+) cells using the Confocal Assistance Program (Leica Confocal Systems, TCS SP8).

### 2.11. Immunohistochemistry

We assessed the number of nigral neurons in diaminobenzidine (DAB) TH-immuno-stained slices of the SNc. First, we washed the slices 3x with PBS +0.5% Triton X-100 for 5 min. Subsequently, we incubated the slices in PBS-SDS 0.5% for 5 minutes at RT. We depleted the endogenous peroxidase by incubating the slices in an isopropanol-peroxide solution (0.3%–10% in PBS. 30 min at RT). We blocked unspecific binding sites by incubating the slices in a solution of PBS containing 0.5% Triton X-100 and 10% normal goat serum (Vector Laboratories; Burlingame, CA, USA, Cat # S-1000) for one *h* at RT, followed by three washes (0.025% PBS-Triton for five min each). Then, we incubated the slices in a rabbit anti-TH polyclonal antibody (1 : 1000 dilution; Chemicon International; Temecula, CA, USA, Cat #T8700) in 2% goat serum and 0.025% PBS-Triton for 48 h at 4°C. Subsequently, we washed the slices and incubated them in a goat anti-rabbit IgG biotinylated (1 : 200 dilution; Vector Laboratories; Burlingame, CA, USA, Cat # BA-1000) in a solution containing 2% goat serum and 0.025% PBS-Triton. Then, we washed the slices three times, 10 min each, with 0.025% PBS; afterward, we incubated the slices in a freshly prepared Avidin-Biotin label solution (at least 30 min before use; one drop of Avidin (A) plus one drop of Biotin (B) per 5 mL of 0.025% PBS-Triton). Then, we washed the slices three times (10 min each) with 0.025% PBS-Triton. We detected the peroxidase reaction by diaminobenzidine using the VectaStain Elite kit (Vector Laboratories; Burlingame, CA, USA, Cat # PK-64100). Finally, we incubated the slices in that solution for 2 to 3 min, washed them with PBS 1 M, and then placed them sequentially on glass slides according to Paxinos and Watson [18]. Once on the glass slides, we left the slices dry for about one week at RT. Then, we dehydrated the slices through a series of ethylic alcohols at increasing concentrations (80–100%) for 1 minute at each solution, passed them to xylene (resin solvent), and finally embedded them in the resin synthetic to cover the slides. We visualized TH (+) neurons with an optic microscope (Leica DM1000LED) using a digital DC300 camera (Leica; Cambridge, UK) at 10x. We cut the slices every 150 *μ*m in the rostrocaudal direction of the SNc.

### 2.12. Stereological Counting of Nigral Neurons

We counted the TH (+) nigral neurons using the StereoInvestigator v.11 (MicroBrightField Inc.) software package coupled to a Nikon Labophot-2 Stereology Microscope equipped with a motorized stage (Ludl MAC-2000 model) with a frame-grabber and a CCTV Exwave HAD Sony camera. We delimited the SNc within precise anatomical references from Paxinos and Watson [18]. Then, we counted the neurons using a 40x NA. 0.5 objective with a lens relay 1x/16 for every 10th slice containing the substantia nigra (6-7 slices per rat). The person counting the neurons was unaware of the experimental rat group.

### 2.13. Golgi Method

We manually counted the dendritic spines of the striatal neurons in slices from isolated brains stained using the Golgi technique [30]. We immersed brain slices containing the striatum into a Golgi solution (potassium dichromate 2.7% and osmium tetroxide 0.3%) for 7 days and then immersed them into a 0.75% aqueous solution of silver nitrate for 2 days. The slices (100 *μ*m-thickness) were dehydrated in ethanol (96% and 100%), and after clearing (eugenol and xylol), they were settled on Entellan-covered slides. Dendritic spines along a 10 *μ*m-length of five secondary dendrites out of 10 MSNs of each striatum were counted using the Sholl method. The person in charge of visually counting the dendritic spines was unaware of the experimental conditions.

### 2.14. Data Analysis

We expressed all values as means ± SEM. We used two-way ANOVA and Bonferroni post hoc test to evaluate the effect of the treatment on balance (beam test) and motor coordination (rotarod) over time. We used one-way ANOVA followed by the Tukey post hoc test to assess the effect of the treatment on the total number of TH (+) neurons, BDNF-flag/TH (+) coexpressing neurons, and the number of dendritic spines. We calculated statistical significance using GraphPad Prism version 10.1.2 (GraphPad Software, San Diego, California, USA). *P* < 0.05 was the index of a statistically significant difference. We show the experimental protocol in Figure 1.

## 3. Results

### 3.1. Combined Treatment Effect on Balance and Motor Coordination

**Figure 2:**
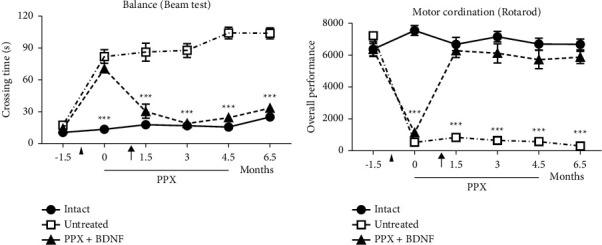
The combined PPX treatment with BDNF-gene transfection restored balance (a) and motor coordination (b). The arrowhead indicated the time of lesion with 6-OHDA. The black arrow indicates the time of BDNF-gene transfection. ^*∗∗∗*^*P* < 0.001 vs. normal and treated rats. Values are means ± SEM. Two-way ANOVA and Bonferroni post hoc test. *n* = 7 each group. Significant recovery was observed 15 days after the BDNF-gene transfection and one month after the PPX infusion.

The loss of balance and motor coordination in PD leads to frequent falls, which cause fractures and complicate the treatment of the disease. We used the beam test to assess balance and motor coordination in rodents [31] to evaluate the effect of the combined PPX + BDNF-gene treatment (Figure 2(a)). The lesioned rats markedly increased the time to cross the length of the beam, and some of them could not cross it in the 2 min allowed to do it (see Figure 2(a)). However, the combined PPX + BDNF-gene treatment completely recovered the time to cross the beam, with crossing times not significantly different from those of intact rats (Figure 2(a)). The first evaluation was done five days after the BDNF-gene transfection and one month after the PPX infusion when the treated rats recovered their initial crossing time. The recovery to typical values seems to be long-lasting because it was present two months after the end of the treatment (Figure 2(a)).

As the beam test, the rotarod performance also assesses motor coordination [32]. The bilateral lesion produced a total loss of motor coordination. The lesioned rats could not remain on the rotating rod, falling from the rod at low rotating speeds (see Figure 2(b)). As in the beam test, the combined PPX + BDNF-gene treatment completely recovered motor coordination evaluated by the rotarod test. The recovery followed similar times to those for the improvement in the beam test (Figure 2(b)). As in the beam test, the recovery may have been permanent because it was present two months after the withdrawal of the treatment (Figure 2(b)).

### 3.2. Normalization of Gait

**Figure 3:**
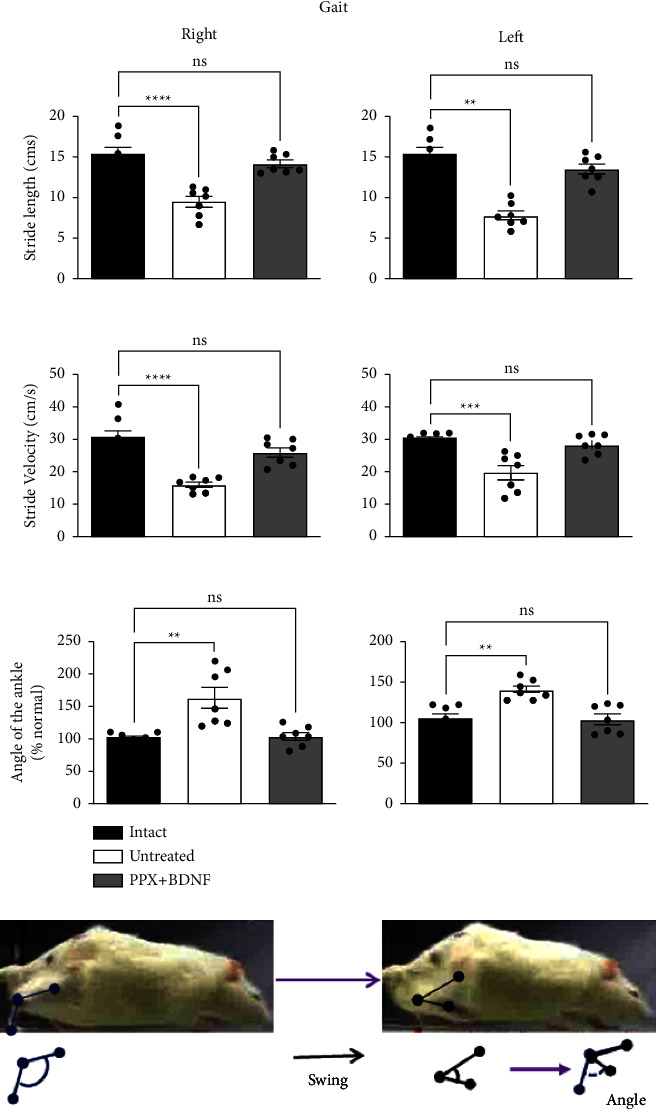
The combined treatment (PPX + BDNF) restored gait. Gait recovery was estimated by the recovery of the stride length (a), stride velocity (b), and angle of the ankle (c) during the swing phase of gait. Data correspond to gait determination at 34 weeks after the dopaminergic lesion, two months after the end of the treatment. Pictures represent changes in the angle of the ankle during the swing. Changes in values are expressed as means ± SEM. ^*∗∗*^*P* < 0.01, ^*∗∗∗*^*P* < 0.001, ^*∗∗∗∗*^*P* < 0.0001; ns, no significant difference. *n* = 7. Data were analyzed by one-way ANOVA followed by Tukey post hoc test.

The bilateral lesion of the dopaminergic nigrostriatal system reduced both stride length (shortened steps) and velocity (bradykinesia) by 40% when compared with the intact group (Figures 3(a) and 3(b)). It also reduced the angle of the ankle (reduced dorsiflexion) during the unrestrained gait of the rats (Figure 3(c)). The changes in gait parameters evoked by the lesion persisted throughout the experiment, indicating no spontaneous recovery. Furthermore, the long-term PPX infusion treatment combined with the BDNF-gene transfection improved the length and velocity of strides and the ankle angle to reach values equal to those of the healthy control group (Figures 3(a)–3(c)). The restoration of the gait parameters persisted two months after the treatment ended, indicating that the recovery was probably permanent.

### 3.3. Rescue of Working Memory

**Figure 4:**
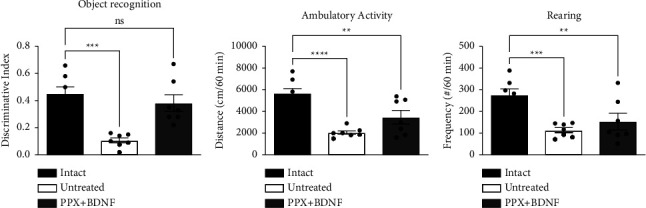
The combined treatment recovered the object recognition index (a) but did not restore rearing or spontaneous ambulatory activity (b, c). Data correspond to gait determination at 34 weeks after the dopaminergic lesion, two months after the end of the treatment. Values are expressed as means ± SEM. ^*∗∗*^*P* < 0.01, ^*∗∗∗*^*P* < 0.001, ^*∗∗∗∗*^*P* < 0.0001; ns, no significant difference. *n* = 7. One-way ANOVA followed by Tukey post hoc test.

One of the nonmotor symptoms experienced by patients with PD is reduced cognition, expressed by reduced working memory. Using the object recognition test, we evaluated the effect of the combined treatment of PPX associated with the BDNF-gene transfection on the working memory of lesioned rats. Dopamine depletion reduced the object recognition index (ORI) by 78% compared to normal rats (Figure 4(a)). By contrast, the rats that received the combined PPX + BDNF-gene treatment fully recovered recognition index values equal to those of normal rats (Figure 4(a)). This result indicates that the combined treatment restored working memory loss because of dopamine depletion.

Furthermore, both ORI reduction and recovery appear to have been permanent since they persisted two months after the end of the treatment. Concerning the exploratory behavior of rats, the bilateral lesion of the dopaminergic nigrostriatal system reduced the frequency of rearing (Figure 4(b)) and ambulatory activity (Figure 4(c)) compared with those of normal controls. In this case, the combined treatment of PPX + BDNF-gene did not evoke any recovery of the rearing or spontaneous ambulation in the lesioned rats. The test was made two months after the end of the drug treatment.

### 3.4. Recovery of TH (+) Neurons

**Figure 5:**
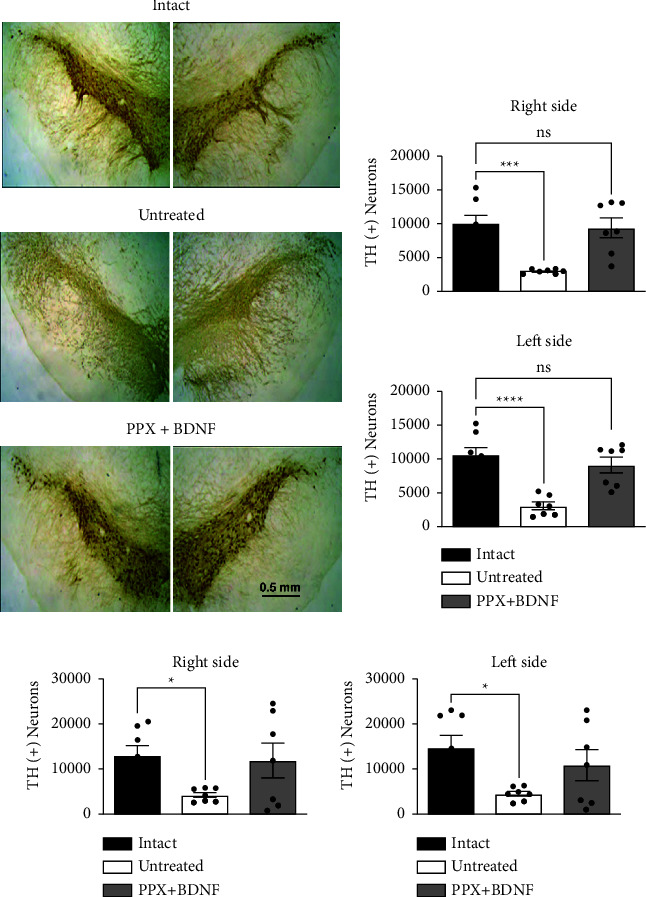
The combined treatment restored the number of TH (+) cells in the substantia nigra compacta and ventral tegmental area. Representative micrographs of TH (+) immunoreactivity (a). Bar graphs of the quantification of TH (+) neurons in the substantia nigra (b) and ventral tegmental area (c). The values are means ± SEM. *n* = 7 (8 sections per rat). ^*∗*^*P* < 0.05, ^*∗∗∗*^*P* < 0.001, ^*∗∗∗∗*^*P* < 0.0001; ns, no significant difference. *n* = 7. One-way ANOVA followed by Tukey post hoc test.

PD [33] and experimental parkinsonism induced by the intrastriatal administration of 6-OHDA [32] have the loss of dopamine neurons in the SNc and VTA in common. The bilateral intrastriatal application of 6-OHDA significantly reduced the number of TH (+) neurons in the SNc and VTA (Figures 5(a)–5(c)). TH (+) neuron number reduction was 65–68% on the right and left side of the SNc (Figure 5(b)) and 68–70% on the VTA of both sides (Figure 5(c)). In contrast, the combined PPX + BDNF-gene treatment fully restored the total number of TH (+) immunoreactivity on both sides of both the SNc (Figure 5(b)) and the VTA (Figure 5(c)). The counting of the neurons was two months after the end of the treatment.

### 3.5. Recovery of Dendritic Spines

**Figure 6:**
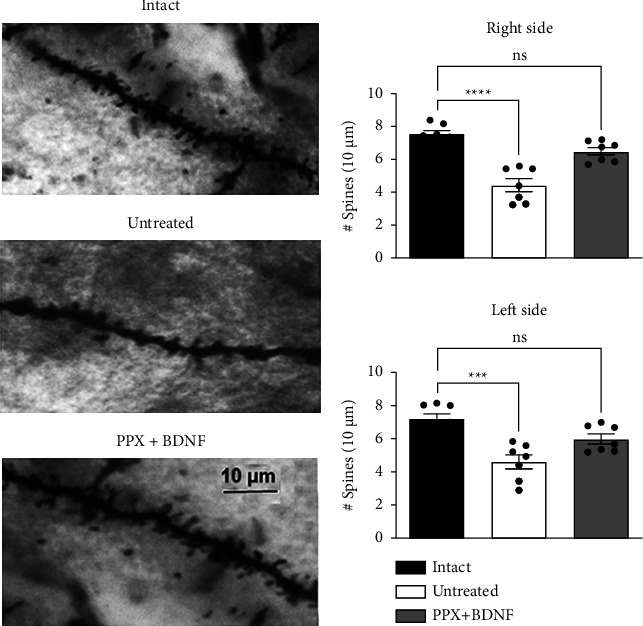
The combined treatment restored the number of dendritic spines of striatal spiny neurons. (a) Illustration of representative experiments. (b) Bar graphs of the number of spines in the distinct groups. The spine count was performed 36 weeks after the lesion in the lesioned and untreated group and two and a half months after the end of treatment in the PPX + BDNF group. The values are means ± SEM. *n* = 7 (8 sections per rat). ^*∗∗∗*^*P* 0.001; ^*∗∗∗∗*^*P* 0.0001. *n* = 7. One-way ANOVA and Tukey post hoc test.

It is known that both PD [1, 2] and experimental PD [6, 7] reduce the number of dendritic spines of the MSNs. The bilateral lesion reduced the number of dendritic spines of the MSNs (Figures 6(a) and 6(b)) by 42% on the right side and 35% on the left side (Figure 6(b)). The combined PPX + BDNF-gene treatment fully restored the number of spines on both sides (Figure 6(b)). The counting of dendritic spines was two months after the withdrawal of the PPX infusion, suggesting that the recovery was permanent.

### 3.6. BDNF-Flag Expression

**Figure 7:**
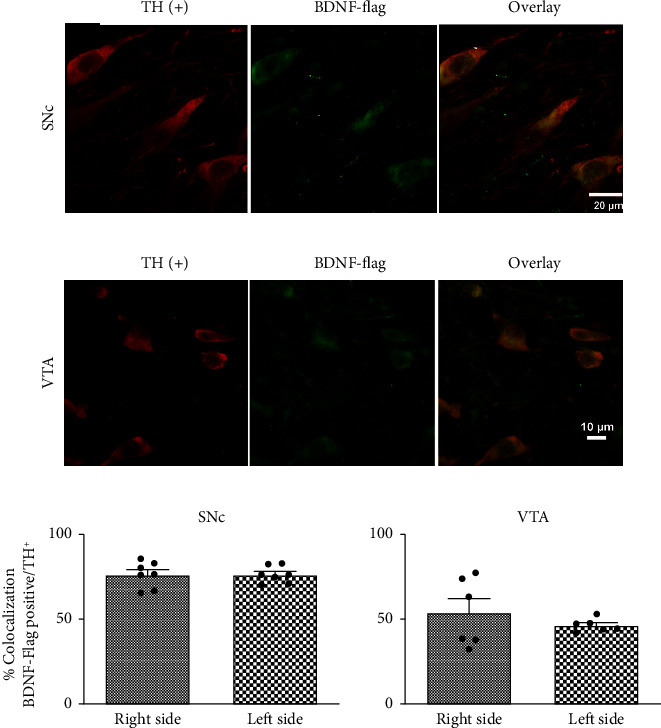
Colocalization of TH+/BDNF-flag in dopamine neurons of the substantia nigra and ventral tegmental area. Representative micrographs (a, b). Immunohistochemistry was performed 36 weeks after the lesion and two and a half months after the end of treatment. The histograms (c) show the percentage of BDNF-flag expression in TH (+) neurons of the substantia nigra *pars compacta* and the ventral tegmental area. *n* = 7.

The bilateral transfection of the BDNF-gene in the SNc using the NTS-polyplex led to the expression of the BDNF-flag in the nigral dopamine neurons, as shown by the double immunofluorescence assays (Figure 7(a)). The expression of BDNF-flag protein was almost 75% in TH-positive neurons on the right and left side of the SNc (Figures 7(a) and 7(c)) and 54 − 50% on the right and left side, respectively, in the VTA (Figures 7(b) and 7(c)). Because BDNF-flag immunoreactivity was detected two months after ending the treatment, the expression of the BDNF-gene was a persistent event after transfection.

## 4. Discussion

The present results show that continuous infusion of the preferential dopamine D3 agonist PPX in conjunction with selective, nonviral transfection of the BDNF-gene to the surviving dopamine neurons restored the number of TH-positive neurons and the spines of striatal neurons. Neuronal and spinal recovery resulted in normalizing motor behavior, as measured by restoring normal gait, motor coordination, and balance. In addition, working memory was restored. Furthermore, the recovery of motor behavior is durable, as it was present two months after discontinuing the selective agonist infusion.

The combined treatment restored the average number of TH-positive neurons in the SNc. It is assumed that a 6-OHDA lesion produces the death of dopaminergic neurons; thus, an almost total recovery number of TH (+) elements is unexpected. There are two explanations for this phenomenon: first, neurogenesis or second, recovery of the TH-positive phenotype in survival neurons that lose it in the lesion. Neurogenesis can be made possible by stimulating D3 receptors during treatment since their behavioral effect soon appears, indicating their activity (Figure 4). If this is the case, no mature striatum innervation can be reached soon. The new neurons and striatal reinnervation should appear speedily and in enough quantity to increase the almost normal number of TH-positive elements and striatal spines required for motor control recovery. On the other hand, nigral neurogenesis is unlikely since it is a fact that remains controversial, and the initial behavioral effect of PPX can also be explained in terms of D3 receptor expression in other brain areas.

The loss of TH phenotype is progressive in the striatal injection 6-OHDA lesion model. It induces first damage of terminals followed by a progressive loss of TH-positive neurons stabilized at 50–70% over 8 to 16 weeks postlesion [34]. It has been observed that a combined cell death and loss of TH-positive staining occur in this model. Thus, the combined treatment can recover neurons that lose the TH phenotype. It is possible since, in rat embryonic cerebral neurons, both BDNF and dopamine induce the dopaminergic phenotype and survival of neurons [12, 13, 16], and the recovery of TH-positive neurons in the SNc and VTA was associated with a high percentage of TH (+) colocalizing the BDNF-flag protein. In conclusion, we felt that combined treatment produces a recovery of the phenotype.

We found two interesting data related to transfection. First, a set of TH-positive neurons did not express the BDNF flag; this can be related to the transfection efficiency since, as in other reports, similar transfection efficiency was reported with this method [17, 24]; this is possible given that the expression of neurotensin receptors is not similar in all the neurons by the lesion. Second, although the NTS-polyplex carrying the BDNF-flag gene was injected into the SNc, the BDNF-flag protein was also present in the neurons of the VTA (Figures 7(b) and 7(c)). The BDNF-flag may have reached the neurons of the VTA through paracrine propagation of the BDNF-flag secreted by the nigral neurons [35, 36].

The dendritic spines of MSNs play a crucial role in processing motor behavior as they are the locus of the corticostriatal and nigrostriatal synapses [37, 38]. The maintenance of the spines requires a continuous supply of BDNF [2]. Postmortem studies in PD patients have revealed reduced MSN spine density and dendritic length [3–5]. Similar morphological changes in MSNs are observed in animal models of parkinsonism [7, 9, 39], probably due to the reduction in the delivery of BDNF by nigral neurons since the striatal neurons do not express neurotrophin [40] but rather through nigral neurons [41] and transported to the striatum [42]. Loss of nigral neurons in PD reduces BDNF content in the substantia nigra and striatum [42]. In our model, it is possible that by restoring nigrostriatal innervation, most BDNF is released into the striatum, restoring the average number of dendritic spines that are associated with the recovery of normal motor behavior. It should be emphasized that for a complete recovery of motor behavior, a complete recovery of dendritic spines should be achieved; thus, it is easier to get it if we assume a recovery of the TH (+) phenotype.

Although the infusion of a D3 agonist alone resulted in a significant recovery (83%) of the number of dendritic spines, allowing recovery of aspects of motor behavior such as motor coordination (rotarod), it did not restore or eliminate gait muscle rigidity [17]. In the present case, the full recovery of the dendritic spines ensured the restoration of all motor circuits that control normal motor behavior. Furthermore, the restored spines should reappear in their original place before disappearing [43], restoring the motor circuits. Of note, no dyskinesias were observed during or after combined treatment, suggesting normal synaptic plasticity of MSNs, which contrasts with the reported increase in density and spine head area associated with the development of levodopa-induced dyskinesia [44–46] or with dopaminergic grafts in the striatum, which can induce dyskinesias [47, 48]. As PD, the bilateral lesion of the nigrostriatal innervation in the rats reduced working memory, as assessed with the object recognition test [49]. The combined treatment restored working memory (Figure 4(a)). This suggested that this process is dependent on dopamine and likely to be present striatally, particularly in the caudate nucleus [50]. Thus, the dorsal striatum recovery of synaptic plasticity by treatments impacts cognition [51, 52], which is also controlled by dopamine [53]. The recovery of motor behavior induced by the combined treatment might last due to the continuous expression of BDNF, as suggested by the presence of the BDNF-flag protein two months after the end of the experiment. Interestingly, neither rearing nor spontaneous ambulatory activity can be normalized by combined therapy. Although this can be attributed to anxiety and a lack of the anxiolytic effect of treatment, more experimentation is required to explain it adequately since anxiety behavior in the model can involve other structures and transmitters as well as basal ganglia [54, 55]; however, contrasting recovery of motor coordination, balance, and gait recovery maintain animals in a good health state.

## 5. Conclusions

The present results show that continuous activation of dopamine D3 receptors with PPX, associated with selective BDNF-gene transfection, increases the number of dendritic spines of striatal neurons, restoring nigrostriatal innervation due to the complete recovery of the TH (+) elements in substantia nigra compacta. Three aspects of the present treatment should be highlighted: (1) the recovery of TH (+) neurons of substantia nigra was nearly the normal number; (2) recovery of dendritic spines of striatal neurons was also just a standard number. Additionally, the recovery of both neurons and dendritic spines appears to have been durable, as the recovery of motor behavior was still present two months after the end of treatment; (3) in contrast to dopaminergic transplants [47, 48], the treatment did not induce dyskinesias. The absence of dyskinesias may be due to the absence of an excessive number of dendritic spines [56]. Nevertheless, despite the need for research to establish the mechanism involved in the recovery of TH (+) phenotype vs. neurogenesis, the experimental results are promising for evaluating the treatment in the clinic as it would provide patients with years of a better quality of life. Therefore, the treatment appears to be disease-modifying.

## Data Availability

Data are available on request due to privacy/ethical considerations.
